# Aquatic Exercise Programs for Children and Adolescents with Cerebral Palsy: What Do We Know and Where Do We Go?

**DOI:** 10.1155/2011/712165

**Published:** 2011-11-24

**Authors:** J. W. Gorter, S. J. Currie

**Affiliations:** ^1^CanChild Centre for Childhood Disability Research, McMaster University, 1400 Main Street West, Room 408, Hamilton, ON, Canada L8S 1C7; ^2^Health Sciences Program, McMaster University, Hamilton, ON, Canada L8S4L8

## Abstract

Aquatic exercise programs may be a beneficial form of therapy for children and adolescents with cerebral palsy (CP), particularly for those with significant movement limitations where land-based physical activity is difficult. The most recently published systematic review (2005) on aquatic interventions in children with CP found supportive but insufficient evidence on its effectiveness. The aim of this paper is to review recently published literature since 2005 with a focus on aquatic exercise for children with CP. In total, six new studies were published with a main focus on aerobic aquatic interventions in higher functioning children and adolescents with CP. Swimming is one of the most frequently reported physical activities in children and adolescents with CP. Therefore, information on its safety and benefits is highly needed, for those with more severe CP in particular. Research design issues are discussed to help guide future research and practice.

## 1. Introduction

Cerebral palsy (CP) is the most common childhood-onset physical disability with varied impact on daily activities and participation [1]. Children's and adolescent's levels of mobility at home, at school, and in the community can be best described with the expanded and revised gross motor function classification system (GMFCS E&R). The GMFCS ranges from level I, representing high functioning individuals who are able or have the potential to walk without limitations, to Level V, individuals with very limited self-mobility requiring very high levels of support (see Table 1) [2]. The GMFCS is associated with ability but does not indicate an individual's level of physical activity or participation [3]. Children and adolescents with CP across the severity spectrum are more likely to have decreased physical activity levels than their peers; thus they are at risk for other negative health implications such as obesity [4] and cardiovascular risk [5]. 

There is potential for aquatic exercise programs to significantly benefit this population [6, 7]. The unique properties of water provide a desirable environment for children and adolescents with CP [8]. For example, weight-bearing requirements, the amount of trunk control, joint load, and effects of gravity are reduced in water [8]. As a result, aquatic physical activity is more protective of joint integrity than land-based activity [9]. Studies have reported that performing motor skills in the water can potentially increase confidence and lead to less resistance to try difficult tasks compared with land training [10]. Furthermore, activities in the water can be fun and more novel for children, potentially enhancing motivation and interest [11]. Aquatic physical activity may be significantly beneficial for higher GMFCS levels, that is, those with significant movement limitations for whom land-based physical activity may be difficult and limited [6]. It should be noted that there are limited land-based programs for this population [12].

Since aquatic facilities are available and public acceptance is high, there is significant potential for aquatic programs to benefit children and adolescents with CP and other populations across the severity spectrum [9, 13]. In 2010, Brunton and Bartlett described exercise participation of adolescents with CP [14]. Swimming was consistently rated as one of the most frequent activities reported by participants; it was the second and third most frequent activity for GMFCS levels I, II, and III, and more significantly, the most frequent activity for higher GMFCS levels IV and V. Similarly, Zwier et al. reported that swimming was the second most frequent activity in children with CP aged five to seven, with 71% of these children reporting participation in swimming [15]. In summary, aquatic activities may be a beneficial form of exercise and physical activity for individuals with CP throughout the lifespan. Furthermore, there is evidence that this population with a range of physical and cognitive abilities is already taking part in aquatic activities.

 However, there is a lack of aquatic activity programming for this population, and thus the effectiveness of such interventions for persons with CP has not been well evaluated [6]. Kelly and Darrah reported in 2005 that despite many observed benefits of aquatic exercise such as improvements in flexibility, respiratory function, muscle strength, gait, and gross motor function, little research has been done on the effects of aquatic exercise [6]. The authors included three papers in their review, but the information was limited by weak methodological rigour. They concluded that “further evidence is needed regarding the effects of aquatic exercise on fitness and its place in physical management programs of children with CP” [6]. Several studies have been published since 2005; thus, it was appropriate to summarize the new research and revisit the findings of Kelly and Darrah. 

This review examines the recent literature (August 2005–January 2011) in a population, intervention, control, and outcome (PICO) fashion. The following specific questions were addressed (1) What is the main focus of current research in aquatic exercise interventions in children and adolescents with CP? (2) What future directions are beneficial for this area of research to move forward?

## 2. Methods

### 2.1. Framework

**Figure 1:**
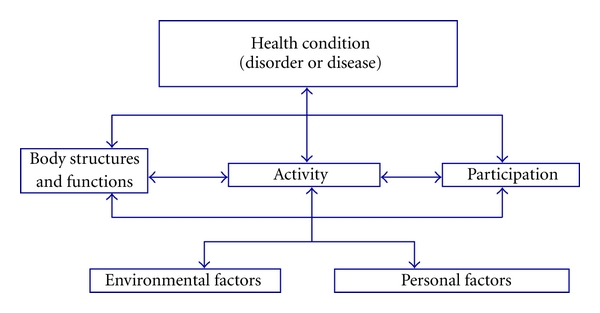
World Health Organization model of the international classification of functioning, disability and health for children and youth (ICF-CY).* Body functions* are physiological functions of body systems (including psychological functions). *Body structures* are anatomical parts of the body such as organs, limbs, and their components. *Impairments* are problems in body function or structure such as a significant deviation or loss. *Activity* is the execution of a task or action by an individual. *Participation* is involvement in a life situation. *Activity limitations* are difficulties an individual may have in executing activities. *Participation restrictions* are problems an individual may experience in involvement in life situations. *Environmental factors* make up the physical, social, and attitudinal environment in which people live and conduct their lives. *Personal factors* are features of the individual that are not part of a health condition or health state.

The international classification of functioning, disability, and Health for Children and Youth (ICF-CY) framework described by the World Health Organization was used in this paper to classify the impact of health conditions according to the effect on body function and structure, activities, and participation (see Figure 1) [16].

### 2.2. Search Strategy

PubMed and CINAHL were searched under the following key words: (1) “cerebral palsy” in combination with (2) “aquatic” and (3) “exercises.” In the PubMed search, “cerebral palsy” was combined with “aquatic,” and in the CINAHL search “cerebral palsy” was combined with “aquatic exercises,” both in a simple search with all results subject to the following inclusion/exclusion criteria. The search was limited to the English language and full articles published from August 2005 to January 2011were to update Kelly and Darrah's [6] search. Inclusion criteria were population (children and adolescents with CP), intervention (aquatic: aerobic, anaerobic, strength, and other), and outcome (body function, activity, and participation). As well, the published study had to involve an intervention. Studies that included children with CP as well as other conditions were also included when relevant (at least one participant must have CP).

### 2.3. Data Extraction

The included papers were read by the authors, and the data was extrapolated and organized into PICO tables (Tables 2 and 3). Data in the PICO Table 2 describes each study's *population* (diagnosis, age, GMFCS level, and number of subjects), *intervention* (aerobic, anaerobic, strength, other, duration, and frequency), *and control* (control, level of analysis). Data in PICO Table 3 includes each study's *outcome* (body function, activity, and participation). In terms of the intervention component, physical activity was categorized as aerobic, anaerobic, strength, or other. “Aerobic” was considered exercise to improve cardiorespiratory fitness. These were typically performed for a long period of time and included activities such as water walking, swimming lengths, and lengths of kicking. “Anaerobic” activities were short lasting and of high intensity, typically lasting a couple of seconds to two minutes. These included activities such as jumping, jumping jacks, and tuck jumps. “Strength exercise” consisted of aquatic resistive training to facilitate increasing strength of musculature. “Other exercises” included activities that do not fall under any of the above categories (e.g., stretching and aquatic play). With respect to the outcome component, outcome measures were classified according to the ICF-CY categories: body function, activity, and participation [16]. Body function included outcome measures such as energy expenditure index (EEI), muscle strength, range of motion and ventilatory and metabolic measurements. Outcome measures such as gross motor function measure (GMFM), the functional reach test, and timed up and go were considered to measure activity. Participation included measures such as Canadian occupational performance measure (COPM).

## 3. Results

**Figure 2:**
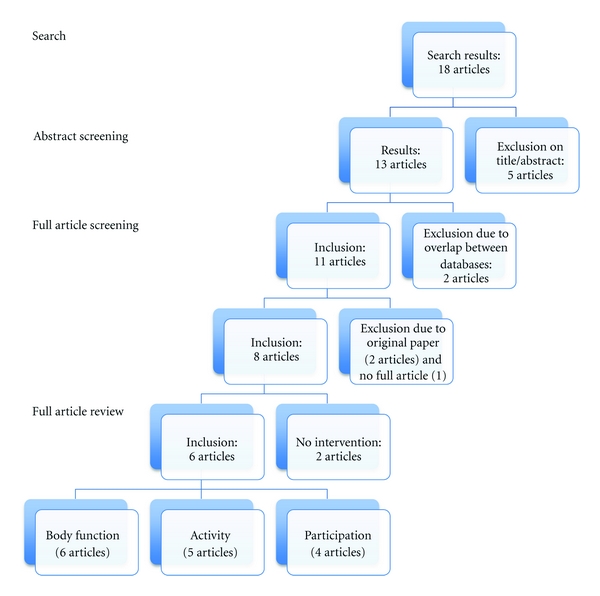
Flow chart.

A total, of 18 articles were collected, twelve of which were excluded as described in detail in Figure 2. In total six articles were selected and included in this paper [7, 9–11, 17, 18].

### 3.1. Population

The population of the six included studies consisted mainly of individuals with spastic CP, specifically spastic diplegia (*n* = 6), hemiplegia (*n* = 5), and quadriplegia (*n* = 2). In addition, two studies included at least one participant with CP and participants with other developmental disabilities and conditions, such as autism, Prader-Willi syndrome, and juvenile idiopathic arthritis [7, 10]. The age range of participants was 2 to 21 years of age, and the number of participants ranged from 1 to 16. With respect to GMFCS levels, the studies included participants with varying levels of functional ability with the following distribution: GMFCS level 1 (*n* = 5), level II (*n* = 4), level III (*n* = 4), and level IV (*n* = 1). None of the studies included participants with GMFCS level V.

### 3.2. Intervention

Of the six studies, all involved aerobic training, [7, 9–11, 17, 18] three anaerobic training [11, 17, 18], three detailed strength training, and three studies were classified as “other” training [7, 9, 10]. Aerobic training included activities such as length swimming, water walking/running, kicking, movement activities in the shallow end, treading water, relay races, and shallow-water aerobics. Anaerobic activities were very limited and included activities such as jumping, jumping jacks, and tuck jumps. Strength or resistance training included using barbells and participating in various lower extremity resistive exercises for hip, knee, and ankle musculature such as latissimus pull downs and wall squats. The interventions ranged from 30 to 60 minutes and were mostly 2 to 3 times per week for 10 to 14 weeks.

### 3.3. Control

None of the six studies used randomization or blinding or had control(s). All of the studies employed a case series design: four studies used ABA design [9, 11, 17, 18], and two AB design [7, 10]. Of the six studies, four analyzed outcome data at an individual level [9–11, 18], one at both an individual and group level [17] and one at a group level [7].

### 3.4. Outcome Measures

Of the six studies, all reported outcome measures of body function. Five studies used mobility-related outcome measures [7, 9–11, 17]. The Canadian occupational performance measure (COPM) was used to measure activities in two studies [10, 18] and participation in another study [11]. One study evaluated self-perception of children and adolescents [9]. Clinically significant improvements have been reported in muscle strength [10], energy expenditure [10, 11, 17], gross motor function scores [9–11], and mobility performance in home, and community environments [7, 10, 11, 18] have been reported.

## 4. Discussion

This paper addressed the focus of current research on aquatic physical activity programs for children and adolescents with CP from August 2005 to January 2011. It was found that the focus of research is on higher functioning children and adolescents with CP, and recent literature still has low internal validity. As well, there is great heterogeneity of intervention and outcome measures, resulting in difficulty in summarizing the findings of these studies.

The majority of these studies focused on populations with ambulatory children and adolescents with spastic CP (diplegia and hemiplegia; GMFCS levels I, II, and III). Only one participant with GMFCS level IV was studied, and none of the studies included individuals with GMFCS level V. Therefore, any interventions using aquatic therapy cannot be generalized to people with more severe motor involvement. As a result, the least is known about the population who potentially may benefit most from aquatic therapy. Water is a gentler environment than land and may allow children with GMFCS levels IV and V especially to exercise in water with more freedom than on land [6]. The feasibility of an aquatic exercise program for children with GMFCS levels IV and V, however, is more difficult than one for higher functioning levels of CP. Personal and environmental barriers such as fear, acceptance, transportation, and accessibility may play a role [19]. Barriers to aquatic physical activity within this population is a topic that was not discussed within the reviewed articles. Thus, it would be beneficial for future studies to report barriers and safety considerations. Of note is the fact that a qualitative study looking at barriers and facilitators of physical activity, including aquatic physical activity, in adolescents with CP is currently underway [20].

The six recently published studies have similar methodological limitations as reported in Kelly and Darrah's review in 2005 [6]. The studies have relatively low sample sizes with a range from 1 to 16 participants, with the majority of studies including less than seven participants and one single-subject study [11]. This impacts the methodological rigour and increases type I error (false positives) [21]. There still is a need for well-designed intervention studies with adequate sample sizes in a population with a broader range of severity levels, including GMFCS level IV and V. It might be useful to recruit and stratify participants by their functional level or baseline physical activity level instead of the traditional markers such as diagnosis, motor impairment, and limb distribution [22]. 

The majority of studies included in this paper involved aerobic aquatic interventions, with an equal distribution of anaerobic, strength, and other interventions across the remaining studies. All of the studies involved an aerobic component. The effectiveness of anaerobic activities for this population was not commented on in the studies and requires further investigation. Since CP causes a permanent disorder of movement and posture [1], it is important for training programs to have a significant muscle strength component to increase postural stability and prevent secondary musculoskeletal impairments [23]. If muscle strength can be increased in the water, it is hoped that this may translate to improved movement on land and in turn increase functional ability. However, there is limited evidence in land-based programs that strength improvements correlate to improvements in activity, as the carry-over effect is generally low or absent [23]. Thus, further research on the carry-over effect from the aquatic environment to activity on land is required. 

The interventions typically lasted for 45 minutes and were run two to three times a week for 10 to 14 weeks. The National Strength and Conditioning Association (NSCA) provides general youth resistance training guidelines that outline resistance training should begin with two to three times per week on nonconsecutive days [24]. A program held two to three times per week allows for adequate recovery between sessions and is effective for increasing strength and power in children and adolescents [24]. Evidence shows that training once per week may be insufficient for enhancing muscle strength in youth. However, this level of exercise may be effective in maintaining the gain in strength following resistance training [24]. In summary, a combination of aerobic and strength exercise may be most beneficial for this population by improving both endurance and muscle strength. 

All of the studies employed a case series design with a majority using an ABA design. Although this study design has its limitations (e.g., lack of control group), the studies that used ABA designs essentially were controlled within the subject through baseline measures. As for data analysis, the majority of studies analyzed data at an individual level. This is usual practice for such studies, as they involved low sample sizes and high heterogeneity of participants. 

The outcome measures of most of the studies focused on body function and activity. The most common measure of function was energy expenditure index (EEI). With respect to activity measures, the majority was standardized activity such as the GMFM, measuring capacity as opposed to capability or performance [25]. There is significant heterogeneity across outcome measures, which results in difficulty conducting a meta-analysis or knowledge synthesis. The use of more generic performance measures in combination with specific individual measures such as goal attainment scaling (GAS) and children's assessment of participation and enjoyment (CAPE) would provide direction and allow for more comprehensive analysis in this field of study. 

There are several potential limitations worth mentioning with the current paper. Only PubMed and CINAHL were searched, excluding others such as MEDLINE, EMBASE, Sports Discus, Cochrane, and PEDro. Therefore, the search was not systematic, potentially limiting the number of studies in this paper. For example a recent publication by Fragala-Pinkham et al. [26] on aquatic exercise programs for children with disabilities was not included although it provided additional information about the study published in 2008 by the same author [7]. Another limitation, as with all reviews, is publication bias: failure to report or publish studies with negative results, which may result in misleading results of reviews that fail to include unpublished studies [21]. Furthermore, the methodological quality of studies was not considered in the inclusion criteria. Thus, studies with poor methodological quality and very low sample sizes were included, increasing the probability of reporting false positives. It was necessary to include these studies, however, due to the limited amount of research in this area.

## 5. Future Directions

In future research, a wider population including several types of CP and higher GMFCS levels, IV and V, should be studied. Furthermore, research on how to overcome the barriers to participation in aquatic programs for this population would be beneficial to move the field forward [21]. Research regarding the minimal intensity levels, frequency and duration to effect change in this population is also required [9, 23]. This will assist therapists in designing a plan of care with the appropriate intervention dosage [10]. 

The American Academy of Pediatrics (AAP) recommends an exercise heart rate exceeding 150 beats to minute to alter aerobic parameters in typically developing children [18]. However, this information is not known for children with CP. Future research would benefit from establishing feasible and practical outcome measures in the water [17]. Ballaz et al. noted difficulty with recording heart rate measurements using a chest belt during swimming activities in adolescents with CP [17]. In the intervention, many recordings were uncompleted due to chest belt displacement or because of floatation belt interferences [17]. Other understudied areas that would benefit from further research include the effectiveness of anaerobic activities for this population, the translation of aquatic outcomes into improvements on land, and the psychological outcome of aquatic physical activity for children and adolescents with CP. There is supportive evidence that aquatic exercise in a group environment can provide a motivating and socially stimulating environment for children [6]. As such, further research regarding outcome measures to assess the psychological effects of aquatic exercise would be beneficial. Except for two studies [17, 26] that commented on the absence of injuries and adverse effects during the study, the safety or risk associated with aquatic physical activity for this population was not measured systematically. Future intervention studies should comment on safety considerations in detail as well as the presence of any adverse outcomes during or after the interventions. Lastly, it would be interesting and beneficial to investigate the possibility of a dose-response effect for aquatic exercise within this population. This would investigate whether there are more improvements and a larger response in outcome measures in physical activity programs with high frequency, duration, or intensity but could also give insight in the uptake and usage of this type of programming among children and adolescents with CP and their families.

## 6. Conclusion

In conclusion, the research evidence on safety and effectiveness of aquatic exercise in children and adolescents with CP is limited and has not significantly changed since the 2005 publication by Kelly and Darrah. There is a strong potential for aquatic physical activity to benefit children and adolescents with CP; however, future studies should involve participants across the GMFCS spectrum with a focus on activity and participation outcomes as well as safety.

## Figures and Tables

**Table 1:** Gross motor function classification system (GMFCS E&R) levels for children with cerebral palsy 6–18 years [2].

Level	Description
I	Walks without limitations
II	Walks with limitations
III	Walks using a hand-held mobility device
IV	Self-mobility with limitations; may use powered mobility
V	Transported in a manual wheelchair

**Table 2:** Summary of findings of the selected intervention studies (population, intervention, and control).

*Study*						
First author	Fragala-Pinkham	Thorpe	Fragala-Pinkham	Retarekar	Ballaz	Kelly
Year	2008	2005	2009	2009	2010	2009
Reference no.	[7]	[9]	[10]	[11]	[17]	[18]
*Population*						
Number	16 (2 CP)	7	4 (2 CP)	1	12	5
Age range (in years)	6–12	7–13	2–19	5	14–21	9–11
CP subtype	Spastic	Spastic	Spastic	Spastic	Spastic	Spastic
Limb distribution	Hemiplegia Diplegia	Hemiplegia Diplegia	Hemiplegia Diplegia	Diplegia	Hemiplegia Diplegia Quadriplegia	Hemiplegia Diplegia Quadriplegia
GMFCS level	I, II	I, II, III	I	III	I, II, III, IV	I, II, III
Other diagnosis	Developmental disabilities		Juvenile Idopathic Arthritis and Prader-Willi Syndrome			

*Intervention*						
Aerobic	Yes	Yes	Yes	Yes	Yes	Yes
Anaerobic				Yes	Yes	Yes
Strength	Yes	Yes	Yes			
Other	Yes	Yes			Yes	
Duration	14 weeks	10 weeks	6 weeks–8 months	12 weeks	10 weeks	12 weeks
Session	45 minutes	45 minutes		30 minutes	45 minutes	60 minutes
Frequency	2 × week	3 × week	1-2 × week	3 × week	2 × week	3 × week

*Control*						
Design	AB	ABA	AB	ABA	ABA	ABA
Analysis	Group	Individual	Individual	Individual	Individual/group	Individual

**Table 3:** Summary of outcomes measures used in the selected studies according to ICF-CY domain (clinically significant changes in bold).

*Study*						
First author	Fragala-Pinkham	Thorpe	Fragala-Pinkham	Retarekar	Ballaz	Kelly
Year	2008	2005	2009	2009	2010	2009
Reference no.	[7]	[9]	[10]	[11]	[17]	[18]
*Outcome*						
Body function	Yes	Yes	Yes	Yes	Yes	Yes
	Muscle strength	EEI Muscle strength, gait velocity	**EEI**, muscle strength (**MMT**, HHD), ROM, pain scale	**Modified EEI**	**EEI**, Muscle strength (knee) Gait	EEI PedsQL-FS
Activities	Yes	Yes	Yes	Yes	Yes	Yes
	**Half-mile walk/run**, modified curl-ups, FTS 3-meter test, Mobility-PEDI	**GMFM-88 (Dimension E)** **TUG** GMFM- (Dimension D) FRT	**GMFM-66** **PEDI** FRT Timed SLS FTS OGS **COPM**	**GMFM-66** 6MWT PAQ	GMFM-88 (Dimension D & E)	**COPM**
Participation		Yes		Yes		
		SPS for children and the SPS for Adolescents		**COPM**		

COPM: Canadian occupational performance measure, EEI: energy expenditure index, FRT: functional reach test, FTS: floor to stand, GMFM: gross motor function measure, HHD: hand-held dynamometer, MMT: manual muscle testing, OGS: observational gait scale, PAQ: physical activity questionnaire, PEDI: pediatric evaluation of disability inventory, PedsQL-FS: pediatric quality of life multidimensional fatigue scale, ROM: range of motion, SLS: single limb stance, SPS: self-perception scale, TUG: timed up and go test, 6MWT: 6-minute walk test.
